# Column setting and text justification influence return-sweep eye movement behavior during Chinese multi-line reading

**DOI:** 10.1186/s41235-024-00559-5

**Published:** 2024-06-03

**Authors:** Mengsi Wang, Donna E. Gill, Jeannie Judge, Chuanli Zang, Xuejun Bai, Simon P. Liversedge

**Affiliations:** 1 Key Research Base of Humanities and Social Sciences of the Ministry of Education, Academy of Psychology and Behavior, Tianjin Normal University, Tianjin, China; 2 Faculty of Psychology, Tianjin Normal University, Tianjin, China; 3 Tianjin Social Science Laboratory of Students’ Mental Development and Learning, Tianjin, China; 4 School of Psychology and Humanities, University of Central Lancashire, Preston, UK

**Keywords:** Chinese multi-line reading, Return-sweeps, Column setting, Text justification

## Abstract

People regularly read multi-line texts in different formats and publishers, internationally, must decide how to present text to make reading most effective and efficient. Relatively few studies have examined multi-line reading, and fewer still Chinese multi-line reading. Here, we examined whether texts presented in single or double columns, and either left-justified or fully-justified affect Chinese reading. Text format had minimal influence on overall reading time; however, it significantly impacted return-sweeps (large saccades moving the eyes from the end of one line of text to the beginning of the next). Return-sweeps were launched and landed further away from margins and involved more corrective saccades in single- than double-column format. For left- compared to fully-justified format, return-sweeps were launched and landed closer to margins. More corrective saccades also occurred. Our results showed more efficient return-sweep behavior for fully- than left-justified text. Moreover, there were clear trade-off effects such that formats requiring increased numbers of shorter return-sweeps produced more accurate targeting and reduced numbers of corrective fixations, whereas formats requiring reduced numbers of longer return-sweeps caused less accurate targeting and an increased rate of corrective fixations. Overall, our results demonstrate that text formats substantially affect return-sweep eye movement behavior during Chinese reading without affecting efficiency and effectiveness, that is, the overall time it takes to read and understand the text.

## Significance statement

The findings from this study shed light on the impact of text format on Chinese reading behavior, specifically in terms of return-sweep eye movements (large saccades moving the eyes from the end of one line of text to the beginning of the next). We compared single and double text column formats as well as left- and fully-justified text formats to show these caused differences in the launch and landing position of return-sweeps, as well as the frequency with which corrective saccades occurred. These insights not only contribute to our understanding of return-sweep behavior in Chinese multi-line reading (a currently under-researched aspect of reading), but also have practical implications for publishers and designers seeking to optimize reading experiences for readers. The study demonstrates that format choices produce trade-offs between different aspects of eye movement behavior in reading. Also, we show that return-sweep behavior provides an important index of general reading performance that, to date, has often been neglected. Our results might also inform decision-making in respect of publishing practices. Text format decisions have significant consequences for reading behavior and here we demonstrate what some of these consequences are.

## Introduction

Over the past five decades, numerous empirical studies have argued that eye movements reflect moment-to-moment cognitive processing during reading (e.g., Liversedge & Findlay, 2000; Rayner, 1998; Starr & Rayner, 2001). The evidence supporting this view largely derives from studies examining eye movements that occur during the reading of single-line sentences rather than multi-line texts, and this is the case even though people spend most of their time reading multi-line texts. In fact, we are consistently presented with multi-line texts from a variety of sources ranging from online materials such as advertisements and expository texts to novels and magazine articles. These materials may be presented in different formats that may make reading easier or more difficult; thus, text presentation format should be of interest to publishers and others in relation to whether there are optimal ways of displaying text to readers to facilitate efficacy of reading. We might characterize efficacy of reading in two ways; reading efficiency, that is how rapidly readers are able to process the information that they are reading, and reading effectiveness, that is how fully they comprehend the information they are reading.

Multi-line text reading requires large eye movements—return-sweeps—to move our eyes from the end of one line of text to the beginning of a new line of text (Hofmeister et al., 1999; Rayner, 1998). A key point here is that return-sweeps have received only limited examination in the literature to date and that formatting design of text may have implications for return-sweep eye movements (Hofmeister et al., 1999; Parker & Slattery, 2019; Slattery & Vasilev, 2019). In the current article, we examined reading and return-sweeps for multi-line texts among Chinese readers focusing on text presented in a single- or double-column format as well as different types of justification to explore the influence on overall reading performance and return-sweep behavior. We considered this would afford us the opportunity of relating issues of text format with indices assessing different aspects of reading behavior, thereby gaining insight into whether text presented in one particular format relative to others might afford a processing advantage to readers. Our focus was on Chinese reading as, among other reasons (see later), Chinese is a language that is used widely across the world, and yet despite this, there has been far less investigation of reading behavior in Chinese than in other alphabetic languages (e.g., English).

### Effects of column setting and text justification on reading

One formatting issue that pertains directly to reading, and specifically, return-sweep behavior in reading is whether text is presented in a single, or in multiple (most often a pair of) columns. Of course, the same amount of text can be presented in a single column or in multiple columns, with the latter format being very common in newspapers and academic articles. In the case of Chinese journals, for example, the main body of text in all academic journals in the field of psychology (according to the “Annual Report for Chinese academic Journal Impact Factors (Social Sciences) 2023”) is presented in two-column format. When a single column is transformed into multiple columns, line length (for each column) is shortened and line number increases to roughly double. Consequently, substantially more return-sweeps are required for the same text when presented in multiple columns relative to single-column presentations.

The influence of column setting on reading performance has generated inconclusive findings to date (for a review, see Tarasov et al., 2015). While some studies have shown that single-column texts with longer lines produce less difficulty in reading compared to multiple-column texts with shorter lines (e.g., Duchnicky & Kolers, 1983; Dyson & Kipping, 1997, 1998; Poulton, 1959), other evidence suggests a reversal of this pattern (e.g., Foster, 1970). Intriguingly, further evidence suggests that differences in reading performance between single-column and multiple-column presentations are not at all evident (Creed et al., 1987; Hartley et al., 1974). However, it is also the case that there was wide variation in several typographic parameters adopted across conditions in these studies (e.g., column width, number of columns, line spacing and even text content), and such confounding factors make it very difficult to clearly ascertain the precise influence of column setting on reading.

Line length, as a major variant in column setting, has attracted more attention from eye movement researchers. Line length has been shown to impact reading such that individuals with poorer reading skills (e.g., dyslexia) benefit from shorter line lengths when reading from a small e-device as evidenced from increased reading speed and less fixations and regressions (Schneps et al., 2013). Of course, presenting text in multiple columns also means that there is less parafoveal information available to the reader on a given fixation. Skilled readers are known to make use of parafoveal information for efficient reading (for a review, see Schotter et al., 2012), and therefore, reduced line lengths (as is the case for text presented in two compared with one column) might act as a constraint on reading. It should be apparent that the precise influence of column setting on reading might not be completely self-evident in that some aspects of the format might facilitate reading, while others might hamper reading.

Another formatting issue that might relate to eye movement behavior in reading is whether text is fully-justified or left-justified. Many multi-line texts appearing in newspapers, books, scientific journals or web pages are presented in fully-justified format. Arguably, fully-justified format offers a neater presentation as both the left and right margins are vertically aligned, thereby giving an impression of uniformity (i.e., a straight vertical edge on both sides of the body of text). In the case of spaced alphabetic languages, when multi-line texts are fully-justified, the uniformity of line length is achieved by introducing variability into inter-word spacing. Furthermore, in some situations it may even be necessary to split a word at the end of a line and adopt a hyphen to signify that the word parts belong together despite portions of the word appearing on different lines of text. In contrast, when texts are left-justified, while the left margin is uniform (as when text is fully-justified), the right margin is ragged and word spacing through the line of text is uniform. Importantly, we note here that the marked and noticeable differences between left-justified and fully-justified text in alphabetic languages like English are much reduced for Chinese texts. The right margin of left-justified texts in Chinese is far less “ragged” than right margins of left-justified texts in word spaced alphabetic languages like English. This is in part due to reduced word length (approximately 72% of Chinese words are two-characters in length, Lexicon of common words in contemporary Chinese Research Team, 2008), but also because written Chinese is character-based and texts in Chinese do not respect line-final word boundaries. This means it is acceptable in Chinese for a two-character word to be split over two lines such that the first character of the word appears at the end of one line, while the second character of the word appears as the first character of the next line of text. In such a situation hyphenation is not adopted meaning that Chinese multi-line texts will usually be formed from as many characters as possible given page restrictions.

Whether one text format is aesthetically more pleasing than another is an issue of personal preference. However, based on early alphabetic reading studies, it appears that fully-justified texts were more difficult to read than left-justified texts as reflected by slower reading rates or reduced reading comprehension scores (e.g., Gregory & Poulton, 1970; Muncer et al., 1986; Sanders & Stern, 1980). It is argued that word spacing variability and unpredictability of word positions in full justification might lead to less efficient reading.

Despite the long-standing research interest in relation to column setting and text justification on reading efficiency, our current knowledge of their influence on online cognitive processing remains equivocal. These discrepancies may arise from two sources. On the one hand, most early investigations were concerned with the legibility of print and only examined reading rate and comprehension scores as measures of reading performance. On the other hand, our increased knowledge about reading processes over the past five decades has predominantly drawn on single-line reading rather than multi-line reading. The equivocality of research findings in this area represents a significant motivation for us to conduct the present study.

Perhaps, the most striking difference between the reading of single-line and multi-line texts is that multi-line reading requires return-sweeps. Return-sweeps have received less attention compared to eye movements that occur in single-line reading. In addition, in multi-line reading studies, fixations immediately preceding or following a return-sweep (about 20% of all fixations, Hofmeister et al., 1999) are usually excluded from analysis (Slattery & Parker, 2019). This is somewhat surprising, considering the prevalence of return-sweeps in natural reading. Accordingly, Suppes (1994) suggested a comprehensive computational model of eye movement control during reading would necessarily need to account for return-sweeps. However, the dominant models of eye movement control in alphabetic reading (e.g., E-Z Reader: Reichle et al., 2003; SWIFT: Engbert et al., 2005; OB1: Snell et al., 2018) have no mechanism to account for return-sweeps. More relevant to the current study, it is also the case that a recent model of eye movement control in Chinese reading also did not include any mechanism to explain return-sweep behavior (Chinese Reading Model: Li & Pollatsek, 2020). These observations do not represent criticisms of current modeling efforts, but instead reflect a notable aspect of eye movement behavior in reading that has been relatively neglected in relation to formal computational modeling accounts.

### Return-sweep and corrective saccades in reading

The launch and landing positions of return-sweeps in alphabetic reading are generally approximately 4–8 letters away from line margins (e.g., Hofmeister et al., 1999; Parker & Slattery, 2019; Parker et al., 2020; Rayner, 1998; Slattery & Vasilev, 2019). As with intra-line saccades, return-sweeps frequently undershoot their intended location of the next line beginning due to systematic and random error (McConkie et al., 1988; see also Cutter et al., 2017, 2018). Given this, it is often the case that when an undershoot happens, a corrective saccade is initiated. In non-reading studies, the speed with which corrective saccades are initiated has been shown to be dependent upon the actual error size, that is, the deviation between the intended and the actual landing position (Becker, 1976; Prablanc & Jeannerod, 1975). A smaller undershoot error is generally associated with a longer correction latency. In contrast, in reading, it remains unclear how the actual size of the return-sweep undershoot might affect correction latency. We will return to this issue in the Discussion.

Paterson and Tinker (1940, 1942) found that reading rate decreased when texts were presented with excessively long or short lines. They speculated that a reduction in reading rate for excessively long lines might be due to readers’ inability to accurately reposition their eyes to the beginning of the following line. In contrast, difficulty in reading very short lines might primarily be due to the unavailability of parafoveal and peripheral information at the line end to allow for pre-processing of text prior to direct fixation. Hofmeister et al. (1999) directly examined how line length of texts affected return-sweep behavior, and found that with increased line length, readers’ eyes landed further away from the left margin and readers initiated more corrective saccades to the left (see also Heller, 1982). This effect has been replicated in recent studies (e.g., Parker & Slattery, 2021; Parker et al., 2019a; Vasilev et al., 2021).

### Return-sweep fixations in reading

The fixation immediately preceding a return-sweep is termed the line-final fixation, whereas the fixation immediately following a return-sweep is called the line-initial fixation. We can classify line-initial fixations as accurate or inaccurate based on the occurrence of a corrective saccade. Furthermore, when the line initial fixation occurs at a point beyond the intended target, it is referred to as an “oversweep-fixation” or an overshoot, whereas when the line initial fixation falls short of its intended target, it is termed an “undersweep-fixation” or an undershoot (e.g., Parker et al., 2017). Note that undersweep-fixations are much more common than oversweep-fixations which occur quite rarely (about 0.97%, Slattery & Vasilev, 2019) during natural reading. Line-final fixations, accurate line-initial fixations, and undersweep-fixations are often compared to fixations that are not adjacent to return-sweeps (referred to as intra-line fixations).

Compared to intra-line fixations, line-final fixations are normally 16 ~ 28 ms shorter (Abrams & Zuber, 1972; Adedeji et al., 2022; Hawley et al., 1974; Heller, 1982; Hofmeister et al., 1999; Rayner, 1977; Rayner, 1978). Rayner (1977) argued that shorter line-final fixations might result from a lack of parafoveal processing. Given that no information is present to the right of line ends, readers have no opportunity to pre-process parafoveal information while the eyes remain positioned at this point. In contrast, Kuperman et al. (2010) suggested that reduced line-final fixation durations might be attributed to readers engaging predominantly in processing associated with oculomotor programming (see also Abrams & Zuber, 1972; Hofmeister et al., 1999; Mitchell et al., 2008; Parker et al., 2019a, 2019b; Parker et al., 2019a, 2019b; Parker & Slattery, 2019; Tiffin-Richards & Schroeder, 2018). In cases where the information at the beginning of the next line of text is quite distant from fixation, readers are very unlikely to obtain useful information from that point (Pollatsek et al., 1993), meaning that the primary processing required during line-final fixations is saccadic programming necessary to redirect the point of fixation to the next useful word in the sentence via a return-sweep (Abrams & Zuber, 1972; Hofmeister et al., 1999).

Accurate line-initial fixations tend to be 30 ~ 50 ms longer than intra-line fixations (Abrams & Zuber, 1972; Hawley et al., 1974; Heller, 1982; Rayner, 1977). Several reasons have been put forward to explain this. During reading, fixation disparity is increased after saccades of greater amplitude (see Kirkby et al., 2008), and thus, disparity is reduced after an intra-line fixation relative to a return-sweep. Stern (1978) suggested that it is this increased binocular disparity after a return-sweep that contributes to an increased line-initial fixation—the idea being that it takes more time to reduce disparity when that disparity is greater than when it is reduced, and this may delay the initiation of subsequent processing (c.f. Kirkby et al., 2008). To examine Stern’s explanation, Parker, Nikolova and colleagues (2019a) recorded binocular eye movements of participants when they read multi-line texts. They reported an increase in the magnitude of binocular disparity at fixation onset following return-sweeps. However, they found that increased magnitude of disparity was independent of the duration of line-initial fixations. Thus, Parker, Nikolova et al. suggested that longer line-initial fixations occur because of a lack of parafoveal processing prior to direct gaze (see also Parker et al., 2017, 2019a; Parker & Slattery, 2019; Rayner, 1977). Finally, Kuperman et al. (2010) and Rayner (1978) also suggested that inflated line-initial fixations might result from the need to rebuild a mode of saccadic programming over the line (see also Pynte & Kennedy, 2006).

Undersweep-fixations are, generally, much shorter than intra-line fixations (being approximately 130 ~ 170 ms vs. 250 ms in duration). Thus, most researchers have assumed that little, or no, lexical processing occurs during undersweep-fixations (Hawley et al., 1974; Shebilske, 1975). Instead, undersweep-fixations have been assumed to be a consequence of oculomotor error, and therefore, the primary objective during undersweep-fixations is to rapidly plan and execute a corrective saccade to the location intended as the target of the return-sweep (Becker, 1976). However, some recent studies have demonstrated that such fixations facilitate processing of the undershot line-initial words. Furthermore, these studies demonstrate that undersweep-fixations facilitate processing of the word on which the original return-sweep lands prior to the corrective saccade. This effect has been termed undersweep pre-processing benefit, and has been shown to occur for both children and young adult readers (Parker & Slattery, 2019; Parker et al., 2020; Slattery & Parker, 2019). This aspect of eye movement behavior has not been investigated in Chinese reading.

### The current experiment

Here, we wished to examine column setting and text justification in Chinese reading as research investigating return-sweeps in non-alphabetic languages is minimal (though see Li et al., 2012). We chose to examine Chinese reading as this is an unspaced language such that spaces or other visual markers between words are absent and hence do not demarcate word boundaries. Therefore, we were able to examine the effects of text justification in the absence of inter-word spacing variability, something that is not possible in most alphabetic languages. Thus, we had an opportunity to investigate the effects of these variables in the absence of a potential confound.

Another characteristic of written Chinese that we considered an advantage for the present examination is the relatively small variation in word length such that approximately 72% of Chinese words are two-characters in length (Lexicon of common words in contemporary Chinese Research Team, 2008). If word length does affect return-sweep eye movement behavior, then we assume that reduced variation in word length should result in a reduced influence on return-sweep saccades.

In the present study, we manipulated column setting (single-column vs. double-column) and text justification (i.e., left-justification vs. full-justification) of Chinese multi-line texts. At a very basic level, it remains an open question as to whether the specific format of a Chinese text impacts the ease with which it may be effectively linguistically processed. And given variability in the nature of previous findings for alphabetic languages, it is difficult to generate precise predictions in respect of whether one format or another will have a disruptive effect on reading (and even more difficult to precisely predict whether any joint effects of column setting and text justification will be additive or interactive). Despite this, it may be possible to generate some (perhaps tentative) a priori predictions on the basis of the main patterns of effects that have been observed.

First, when text is presented with full justification, it has a greater degree of visual regularity than when it is presented with left-justified format (no ragged right edge). In principle, this means that successive return-sweep saccades required for reading could be more uniform in their extent and this might lead to more efficient saccadic programming and execution. In turn, this might result in more efficient reading and readers might launch and land their return-sweep from a position further from the margins with reduced probability of return-sweep undershoot. Also, the regularity of text justification might cause reduced refixation behavior (including reduced numbers of undershoot fixations) which might result in shorter overall reading times.

Next, with respect to column setting, when splitting a single-column text into double columns, the length of the lines is naturally reduced and the total number of lines increases. This means that more return-sweeps are required in the reading of double- compared with single-column texts. If return-sweeps represent an interruption to reading, that is, at some level, cause disruption to processing (even if very temporarily), then overall reading behavior should be less efficient for text set with double, than single, columns. Readers will likely make more fixations of longer duration and take longer overall to read texts with two, than one, column. In addition to the disruptive effect of return-sweeps, readers have little or no preview of the upcoming word prior to the initiation of the return-sweep. The cumulative effect of a lack of preview at line endings may also negatively impact overall reading efficiency. By contrast, the cost of increased return-sweeps in reading double-column texts may be counteracted by more efficient return-sweep targeting (recall that return-sweeps launched from long lines land further from the left margin with more corrective saccades compared to those launched from short lines). If we assume that this holds for double- and single-column text, it is very likely that return-sweeps will land closer to the left margin and that there will be fewer corrective saccades for double- than for single-column text. In turn, therefore, one might anticipate the overall reading time for a two-column text to be comparable to, or even less than, that of a single-column text.

We might also expect to observe interactive effects between column setting and text justification if the disruption associated with return-sweeps for left-justified text is greater than that for fully-justified text, and any such disruption accumulates over return-sweeps. Since readers make approximately twice as many return-sweeps for double- than single-column text, then under an assumption of accumulative effects, any disruption (due to fully- compared with left-justified text) would be multiplicatively greater under double-column relative to single-column format. Alternatively, if modulation of return-sweep disruption by justification occurred and it was non-cumulative, then effects of column setting and text justification would be additive.

We should also consider the relationship between intra-line fixations and return-sweep fixations. In the reading of alphabetic languages, it is well documented that, compared to intra-line fixations, line-final fixations are shorter, accurate line-initial fixations are longer and undersweep-fixations are shortest (Abrams & Zuber, 1972; Adedeji et al., 2022; Hawley et al., 1974; Heller, 1982; Hofmeister et al., 1999; Parker et al., 2020; Parker & Slattery, 2019; Rayner, 1977; Rayner, 1978; Slattery & Parker, 2019). We have no empirical grounds to anticipate that this basic pattern of effects might differ between alphabetic languages and character-based languages, and therefore, we expect that in the reading of Chinese multi-line texts, we might obtain the same pattern of results. However, whether the magnitude of any differences between intra-line fixations and return-sweep fixations might be increased or reduced in Chinese relative to effects observed in alphabetic languages is difficult to predict. It has been demonstrated that Chinese readers make fixations that are longer than those for English and Finnish readers when reading comparably translated text (Liversedge et al., 2016, 2024). The increased fixation durations for written Chinese have been attributed to its increased visual and linguistic density (e.g., Liversedge et al., 2016, 2024). Thus, if differences between intra-line fixation durations and return-sweep fixation durations arise due to differences in linguistic processing, then they might be more pronounced in Chinese than in alphabetic reading. Alternatively, if such effects are a consequence of processing that is primarily visual in nature, then those differences might be comparable, or even somewhat reduced.

These hypotheses reflect format influences on the efficiency of Chinese reading, yet it remains far less clear how any such efficiency effects we might observe co-occur with effects associated with effectiveness of reading (comprehension effects). Based on a broad range of studies investigating eye movements in reading, while we consider it quite plausible that text format might impact aspects of oculomotor behavior, we suspect it will be much less likely that overall comprehension rates will suffer substantially. Nonetheless, as is standard in eye movement studies investigating reading, we did use comprehension questions to ensure that participants understood the texts they read in this experiment, and given this, we also evaluated comprehension performance in respect of text format.

## Method

### Participants

Given the sparsity of studies that have investigated the influence of column setting and text justification on Chinese reading, the most comparable and reliable effect is the line length effect observed in English reading. Vasilev et al. (2021) and Parker & Slattery (2021) have found robust line length effects on return-sweep landing position, with an effect size of 0.62 and 0.81, respectively. We adopted the average value of 0.72 as a prior effect size. A power analysis was conducted using the PANGEA software (Westfall, 2015). The analysis showed that at least 10 participants per condition were required for 16 stimuli to achieve 80% power (Cohen, 1988) in relation to line length effects. In respect of the text justification manipulation here, it was impossible to obtain effect size estimates since no such studies have been conducted in Chinese reading to date.

Forty-four students from Tianjin Normal University participated in the current study (4 men) with a mean age of 21 years (SD = 2.2 years, range: 18–28 years). Participants were native Chinese speakers with normal or corrected-to-normal vision and no history of reading disorders. Participants provided informed consent and were naïve as to the purpose of the study.

### Apparatus

The experiment was programmed with SR Research Experiment Builder Software. An SR Research EyeLink 1000 Plus eye tracker was used to record participants’ eye movements with a sampling frequency of 1000 Hz. Viewing was binocular but only the right eye was recorded. Texts were double-spaced and were presented in Song font size 25.6 in black on a white background on a monitor (width 37.5 cm and height 30 cm) with a screen resolution of 1,280 × 1,024 and a refresh rate of 144 Hz. Viewing distance was 70 cm. From this distance, each character occupied approximately 33 pixels and subtended 0.79 degree of the visual angle.

### Materials and design

**Fig. 1:**
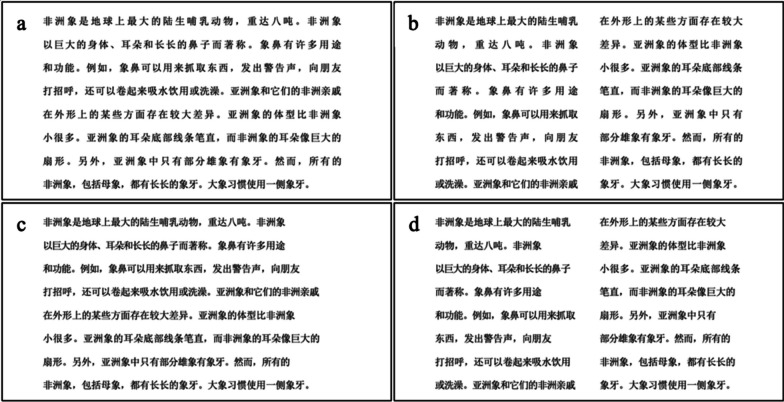
Example stimuli across conditions. Panel **A**: fully-justified text in a single column. Panel **B**: fully-justified text in double columns. Panel **C**: left-justified text in a single column. Panel **D**: left-justified text in double columns. The page of text presented is a part of a passage describing African elephants

Twenty expository texts were selected as multi-line reading materials, and four of them were used for practice. The texts were sourced from the internet and included a range of topics (e.g., photosynthesis, the Trevi fountain, chinchillas). On average, each text contained 483 Chinese characters. When measured in words, each text comprised an average of 282 words (92% of them were one or two characters in length). Passages were split into pages with each one containing 8 or so lines of text (the final page of each passage sometimes had fewer lines than this (27%), but at least 2 or 3). Each text comprised 2–4 pages. In total, there were 48 pages of text in the experiment. The texts were either fully-justified or left-justified and were presented in single-column or double-column format (see Fig. 1). In the double-column condition, the content on each page was identical to that presented in the single-column condition with the exception that the total number of lines was doubled. Importantly, we ensured that the line-final words in the single-column condition appeared at the end of alternate lines in the double-column condition (note again, the content of the sentences was identical across conditions). This afforded us the opportunity of comparing return-sweeps launched from the same line final words and to the same subsequent line initial words across conditions directly. For each text, three comprehension questions were compiled to examine the extent to which participants had understood the text. The questions were presented in an order corresponding to the first third, the second third and the rest of the text’s content.

Recall that, in standard Chinese text, the boundaries of line-final words are not respected in relation to line endings, thereby resulting in some situations in which line-final words would have their constituent characters split over two lines. Li et al. (2012) demonstrated that when words are split across lines in Chinese there is significant disruption to lexical identification, reporting that readers experienced more difficulty processing words presented in this way than when words were presented as a whole (see also Zhang et al., 2022). In the present study, we maintained the integrity of line-final words such that all line-final words appeared in their integrity on the same line, to minimize any potential processing difficulty inherent in splitting words across different lines of text.

To ensure ragged right margins for the left-justified texts, we were careful in the construction of our stimuli, monitoring the length of intra-line words to ensure different line lengths across the single- and double-column conditions. Additionally, we very minimally adjusted spacing between characters in the middle of lines (i.e., that were not adjacent to line-final, or line-initial words). Thus, the texts in our left-justification condition appeared quite natural even though they displayed slightly more ragged right margins than might be observed in standard left-justified Chinese text.

A 2 (column setting: single vs. double) × 2 (text justification: left-justified vs. fully-justified) repeated-measures design was employed. Each participant read twenty texts in total. Four texts for the experiment plus one for the practice per condition. Texts were arranged into four blocks. Each block contained five texts under the same presentation condition. The order of texts was randomized across participants; the assignment of texts to each block and the order of running blocks were counterbalanced using a full Latin square design.

### Procedure

Participants received an information sheet outlining the experimental procedure prior to commencing the study. Once they confirmed that they understood the experimental procedure, a nine-point calibration was performed, and validation error rate was maintained at < 0.5°. During testing, we minimized head movements using a chin rest while participants forehead maintained contact with a sponge strip.

Testing was divided into four blocks. In each block, participants first read one practice text to familiarize themselves with the experimental procedure, following which four experimental texts under the same presentation condition were presented. Participants read 48 pages of experimental texts in total over four blocks. In a single trial, participants read a page of text. At the start of each trial, participants were required to gaze at a black circle on the top left of the screen. Once a stable fixation was obtained, the stimulus (i.e., a page of text) replaced the black circle on the screen. In the event a stable fixation could not be obtained, recalibration was carried out. Participants read silently at their own pace and pressed the space bar to proceed to the next page. A series of three literal “yes/no” comprehension questions followed each text in turn (e.g., Do African elephants weigh up to two tons? Does an African elephant have large fan-shape ears? Does the gestation period of an elephant last 22 months?), and participants indicated their response by pressing one of two keys on the keyboard. The answers to the comprehension questions were an equal number of “yes” and “no” responses. Participants were free to take breaks throughout the experiment, though breaks were encouraged only after completion of a full passage of text. Recalibration was performed after each break and whenever necessary. The whole testing session lasted approximately one hour. Participants were compensated 40 RMB on completion of the experiment.

### Data analysis

Amongst the 48 pages of text for the experiment, six pages that contained three lines or less were removed from data analysis. We scrutinized the raw eye movement data and deleted a total of 138 trials for which the recording quality was poor (7.5% of 1848 experimental trials). Fixations that were shorter than 80 ms or longer than 800 ms were removed using the clean function in Data Viewer. Prior to statistical analyses, we removed data that were outside + / − 3 standard deviations from the mean by participant and condition for each continuous measure that we examined. On average, 2% of the data were removed due to data trimming.

Three sets of analyses were carried out to examine the effects of column setting and text justification. The first set of analyses examined overall reading performance including comprehension accuracy, page reading time, and two global measures of eye movements. Comprehension accuracy is the rate at which participants answered comprehension questions correctly after reading each experimental text. Page reading time refers to the total time taken to read a page of text and press the button to terminate the display. The two global measures we analyzed were average fixation duration (the mean duration of all fixations) and fixation count (number of fixations made on a page). Our second set of analyses examined measures of return-sweep saccade behavior. This focused on return-sweep launch position (the distance from the fixation immediately preceding a return-sweep to the right margin of a line or the right edge of the line-final character) and return-sweep landing position (the distance of the fixation immediately following a return-sweep to the left margin of the new line or the left edge of the line-initial character). We also explored undersweep-fixation location (the distance of the undersweep-fixation to the left edge of the first character of the line). Finally, we examined the frequency of corrective saccades—that is, leftward saccades immediately following an undersweep-fixation (sometimes referred to as return-sweep undershoot error rate). The third set of analyses compared intra-line fixations against return-sweep fixations. In these analyses, we also examined whether our manipulations of text format affected return-sweep fixation durations. We computed line-final fixation durations, the mean duration of the last fixation prior to a return-sweep, the duration of accurate line-initial fixations (fixations after a return-sweep for which there was no immediate leftward corrective saccade). And finally, we computed mean undersweep-fixation durations, namely the mean duration of line beginning fixations after which participants made a leftward corrective saccade. For the return-sweep landing and launch position results, an additional set of analyses was carried out to examine whether the effects of text justification and column setting accumulated over return-sweeps. To do this, order of return-sweeps was included as an extra fixed factor alongside column setting and text justification.

All the analyses were run in the R environment (R Core Team, 2021; R version 4.0.5). For continuous measures, linear mixed-effects models (LMMs) were adopted and fitted using lme4 package (version 1.1–26, Bates et al., 2015). To increase the normality of the data, logarithmic transformation was performed prior to running the LMMs. For binary measures (e.g., accuracy), we used logistic generalized mixed-effects models (GLMMs). In the first instance, each model was constructed with a full random-effects structure (Barr et al., 2013). That is, intercepts and slopes were included for both random factors (i.e., participants and items). For example, the first model fit for a continuous measure for the current study was: lmer (DV ~ Column Setting *Text Justification + (1 + Column Setting *Text Justification |participants) + (1 + Text Justification* Column Setting |items), Data file). Note that the data sets used varied across measures, and therefore, the ‘items’ component in the random-effects structure changed depending on the measure being examined. When the full model failed to converge, we trimmed the full random-effects structure step by step until the model converged successfully. Sliding contrast coding (*contr.sdif* function in MASS package, version 7.3–53.1) was used for the examination of effects of column setting and text justification, where the single-column condition was the baseline for column setting effects and the fully-justified condition was the baseline for text justification effects. Note that, for sliding contrast, the intercept refers to the estimate of the grand mean. Treatment contrast coding was adopted for comparisons between intra-line fixations and return-sweep fixations, where the intra-line condition was the baseline. Regression coefficients (*b*), standard errors (SE), and t/z-values are reported. To compute *p*-values, lmerTest package was run (version 3.1–3; Kuznetsova et al., 2017).

## Results

### Comprehension accuracy, page reading time, and global measures of eye movements

Table 1 shows the descriptive statistics for this set of measures as a function of text justification and column setting. Table 2 shows the corresponding results of GLMM/LMM.

**Table 1:** Means (standard errors) for comprehension accuracy, page reading time (in seconds), average fixation duration (in milliseconds), fixation count as a function of text justification and column setting

	Fully-justified		Left-justified	
	Single column	Double columns	Single column	Double columns
Comprehension accuracy	85%	83%	86%	84%
	(9%)	(10%)	(9%)	(10%)
Page reading time (s)	32.5	31.6	32.7	31.5
	(3.7)	(3.5)	(3.4)	(3.4)
Average fixation duration (ms)	225	224	227	228
	(4.2)	(3.6)	(3.6)	(3.8)
Fixation count	116	114	116	112
	(12.7)	(12.4)	(11.2)	(11.6)

**Table 2:** Fixed effects estimates from the GLMM for accuracy and LMMs for page reading time, average fixation duration, fixation count

Dependent measure	*b*	SE	*t/z*	*P*
Comprehension accuracy				
Intercept	1.89	0.2	9.51	** < .001**
Text justification	0.07	0.12	0.55	.586
Column setting	− 0.11	0.12	− 0.89	.376
Justification*Column	0.02	0.25	0.07	.942
Page reading time				
Intercept	3.35	0.07	45.84	** < .001**
Text justification	0.00	0.02	0.11	.912
Column setting	− 0.02	0.02	− 1.19	.233
Justification*Column	− 0.02	0.06	− 0.42	.674
Average fixation duration				
Intercept	5.41	0.02	332.36	** < .001**
Text justification	0.01	0.00	2.19	**.029**
Column setting	0.00	0.00	0.27	.789
Justification*Column	0.00	0.01	0.08	.932
Fixation count				
Intercept	4.63	0.07	67.64	** < .001**
Text justification	0.00	0.02	0.16	.871
Column setting	− 0.03	0.02	− 1.61	.107
Justification*Column	0.01	0.04	0.17	.864

Fully-justified condition is the baseline for the analyses of the text justification effect. Single-column condition is the baseline for the analyses of the column setting effect. LMM analyses are based on log-transformed data. Significant terms are marked in bold

Mean comprehension accuracy was 84% (SD = 6.4%, Range = 69–98%), indicating that participants understood the experimental texts. The GLMM results demonstrated that comprehension accuracy did not differ between different presentation conditions. This indicated that participants were equally effective at comprehending texts regardless of text justification and column setting. Similar to comprehension accuracy, no significant effects emerged for fixation count or page reading time. For the average fixation duration, the LMM results demonstrated the effect of text justification was significant such that average fixation duration was longer for left- than fully-justified text, though the mean difference (about 3ms) was very minor.

To sum up, the influence of text justification and column setting on the effectiveness and overall efficiency of reading was very limited.

### Return-sweep and corrective saccades

Descriptive statistics for the return-sweep and corrective saccades are presented in Table 3, while fixed-effects estimates from GLMM/LMM are presented in Table 4. Note that, for all these measures, we regarded lines where a return-sweep might occur as the ‘items’ component in the random-effects structure of GLMM/LMM. Recall, we ensured that line-final words appearing in the single-column condition were identical to those appearing in the successive lines in the double-column condition. Thus, we only examined return-sweep and corrective saccades that were directly comparable (i.e., were made between the same words) across conditions. This also applies to the examination of return-sweep fixation durations reported in the next section.

**Table 3:** Means (standard errors) for return-sweep and corrective saccades as a function of text justification and column setting

	Fully-justified		Left-justified	
	Single column	Double columns	Single column	Double columns
Return-sweep launch position (characters)	4.3	2.3	3.5	2.2
	(0.7)	(0.2)	(0.5)	(0.2)
Return-sweep landing position (characters)	3.5	2.4	3.2	2.3
	(0.4)	(0.2)	(0.4)	(0.2)
Undersweep-fixation location (characters)	4.6	3	4.1	2.7
	(0.9)	(0.4)	(0.7)	(0.4)
Frequency of corrective saccades	45%	32%	46%	34%
	(7%)	(6%)	(6%)	(6%)

**Table 4:** Fixed effects estimates from LMMs for return-sweep launch position and return-sweep landing position and GLMM for the frequency of corrective saccades

Dependent measure	*b*	SE	*t/z*	*P*
Return-sweep launch position				
Intercept	0.65	0.04	17.96	** < .001**
Text justification	− 0.11	0.04	− 2.99	**.003**
Column setting	− 0.26	0.04	− 7.06	** < .001**
Justification*Column	0.06	0.04	1.69	.090
Return-sweep landing position				
Intercept	0.76	0.04	18.72	** < .001**
Text justification	− 0.05	0.02	− 1.91	.057
Column setting	− 0.22	0.03	− 7.67	** < .001**
Justification*Column	0.01	0.06	0.12	.902
Undersweep-fixation location				
Intercept	1.00	0.03	32.55	** < .001**
Text justification	− 0.08	0.02	− 3.42	**.001**
Column setting	− 0.30	0.02	− 12.42	** < .001**
Justification*Column	− 0.01	0.05	− 0.14	.891
Frequency of corrective saccades				
Intercept	− 0.49	0.09	− 5.67	** < .001**
Text justification	0.11	0.05	2.35	**.019**
Column setting	− 0.55	0.07	− 7.36	** < .001**
Justification*Column	0.10	0.09	1.06	.288

Fully-justified condition is the baseline for the analyses of the text justification effect. Single-column condition is the baseline for the analyses of the column setting effect. LMM analyses are based on log-transformed data. Significant terms are marked in bold

For return-sweep launch position, the LMM results showed significant main effects of text justification and column setting. Return-sweep launch position was closer to the right margin of lines (the right edge of the line final character) in the left-justified condition than the fully-justified condition and for the double-column condition than the single-column condition. The interaction between text justification and column setting was not significant.

We found a significant main effect of column setting on return-sweep landing positions. Return-sweep landing position was closer to the left margin of a new line (the left edge of the line initial character) in the double-column condition than the single-column condition. The main effect of text justification was not significant though there was a numerical trend suggesting that return-sweep landing position was closer to the left margin of a new line in the left-justified condition than the fully-justified condition. Again, there was no interaction between text justification and column setting on return-sweep landing position. We also examined undersweep-fixation location and found similar results. To be specific, undersweep-fixation location was closer to the left margin of a new line in the left-justification condition than the full-justification condition and closer in the double- than the single-column condition.

For the frequency of corrective saccades, the GLMM results showed robust main effects of text justification and column setting. Readers made significantly more corrective saccades in the left- than the fully-justified condition (40% vs. 38%) and more in the single- than the double-column condition (45% vs. 33%). No significant interaction between text justification and column setting was observed.

To summarize, the results align well with our predictions. Both text justification and column setting had a robust impact on return-sweeps and corrective saccades with additive rather than interactive effects. Return-sweep launch and landing positions were closer to line extremes in the left- than the fully-justified condition and closer in the double- than the single-column condition. The same pattern of results also occurred in relation to the locations of undersweep-fixations. Corrective saccades occurred more often in the left- than the fully-justified condition and more in the single- than the double-column condition.

**Fig. 2:**
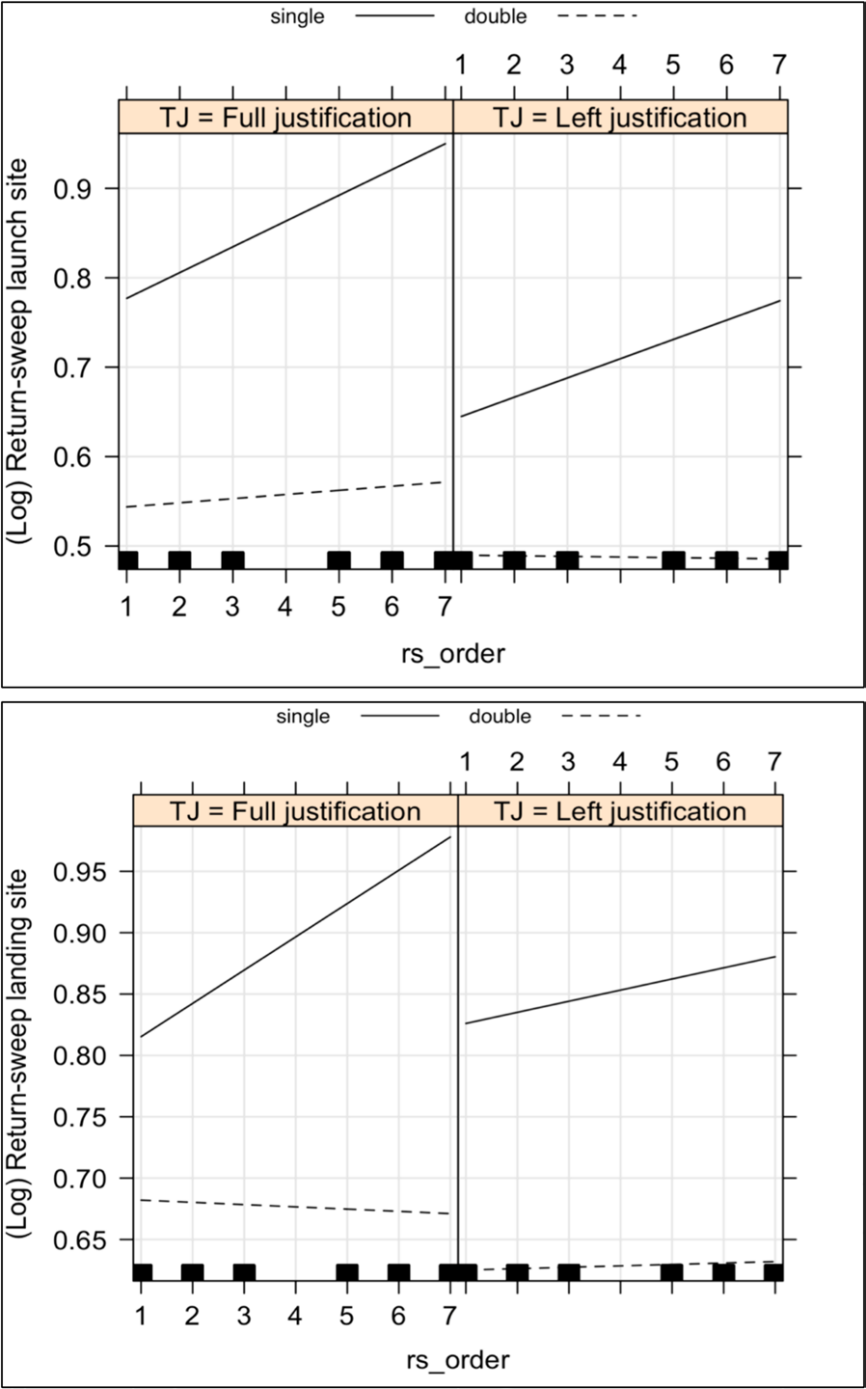
Fixed-effects estimates from the LMMs for log-transformed return-sweep launch position (top panel) and log-transformed return-sweep landing position (bottom panel) as a function of return-sweep order, text justification, and column setting

In a further set of analyses, we explored whether effects of text justification and column setting accumulated over return-sweeps, assessing whether the effects of our variables were additive or interactive in nature. We included the order in which the return-sweeps were made during text reading as an additional fixed factor within the original fixed-effects structure of GLMMs/LMMs. It was anticipated that if effects were accumulative, then there would be a positive correlation of sequential return-sweep order with effect size. Our analyses showed that none of the correlations between text justification and return-sweep order were statistically significant (all *p*s > 0.18), thereby providing no evidence to suggest that our text justification effects were cumulative. In contrast, we found significant interactive effects between column setting and return-sweep order on return-sweep launch and landing positions (*b* =  − 0.02, SE = 0.01, *t* =  − 2.73, *p* = 0.006; *b* =  − 0.02, SE = 0.01, *t* =  − 2.67, *p* = 0.008, respectively). As shown in Fig. 2, for return-sweep launch and landing positions, cumulative effects occurred when the texts were presented in a single column. In contrast, such effects were not evident for texts presented in double columns. These results suggest that as the number of return-sweeps accumulated, return-sweep landing position became increasingly more distant from the left margin for single-column condition. As can be seen from Fig. 2, it is also the case that there is little suggestion of a numerically reduced but comparable effect for text presented in double columns. Quite why this effect only occurs when longer rather than shorter saccades are made is not immediately apparent, though it is possible that because lines of double-column text are approximately half the length of those in single-column texts, there is less opportunity to observe proportionally comparable shortfall under the former than the latter condition.

### Intra-line fixations and return-sweep fixations

Next, we report results of analyses of intra-line fixations and return-sweep fixations including line-final, accurate line-initial fixations, and undersweep-fixations. To reiterate, intra-line fixations were those not temporally contiguous to a return-sweep. Line-final fixations were those immediately preceding a return-sweep, while line-initial fixations were those immediately following a return-sweep. Accurate line-initial fixations corresponded to fixations not followed by a leftward saccade. Undersweep-fixations corresponded to fixations between a return-sweep and a leftward corrective saccade.

**Fig. 3:**
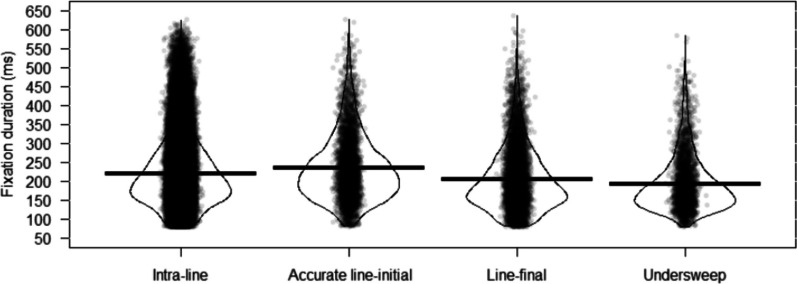
Pirate plots for fixation duration as a function of fixation type. Horizontal solid lines represent the mean per fixation type

First, we compared the duration of intra-line fixations to those of the three types of return-sweep fixations. We did this because we wanted to compare fixation durations at a global level regardless of format. To do this, a variable named ‘fixation type’ was generated to indicate to which type a fixation belonged. We removed all initial fixations occurring in the reading of a page of text from the statistical analyses. Recall that for these analyses we anticipated effects that were comparable to those observed for alphabetic languages, though with potentially minor variability in the magnitude of effects. In the LMM models, fixation type was included as a fixed factor and in the random-effects structure, page of text was regarded as the ‘item’ component. We found a significant difference between intra-line fixation duration and return-sweep fixation duration (see Table 5). As shown in Fig. 3, compared to intra-line fixation duration (*M* = 223 ms, SD = 82), line-final fixation duration was significantly shorter (*M* = 206 ms, SD = 78), accurate line-initial fixation duration was significantly longer (*M* = 235 ms, SD = 78), and undersweep-fixation duration was significantly shorter (*M* = 199 ms, SD = 69). This pattern of effects is, as we predicted, broadly similar to effects observed for alphabetic languages. However, we note that the effect sizes between intra-line and line-final fixation durations (17 ms), accurate line-initial fixation durations (12 ms), and undersweep-fixation durations (24 ms) are reduced relative to those observed in alphabetic languages (22 ms, 35 ms, and 66 ms, respectively). Given this, it is likely that the effects observed here derive from aspects of visual (rather than linguistic) processing.

**Table 5:** Fixed effects estimates from LMMs for fixation duration as a function of fixation type

	*b*	SE	*t*	*P*
Intercept	5.33	0.02	326.21	** < .001**
Line-final fixation	− 0.08	0.00	− 21.79	** < .001**
Accurate line-initial fixation	0.06	0.00	12.73	** < .001**
Undersweep-fixation	− 0.12	0.01	− 19.93	** < .001**

Intercept refers to an estimate mean for intra-line fixation duration. Treatment contrast is used. Intra-line fixation condition is the baseline. Significant terms are marked in bold

Next, we examined whether our manipulations of text justification and column setting affected return-sweep fixation duration.^1^ Consistent with the analyses of return-sweeps and corrective saccades, lines where a return-sweep might occur were treated as the ‘items’ component in the random-effects structure. Descriptive statistics for return-sweep fixation duration as a function of text justification and column setting are presented in Table 6, and the corresponding fixed effect estimates from LMMs are presented in Table 7.

**Table 6:** Means (standard errors) for line-initial, line-final fixations and undersweep-fixations as a function of text justification and column setting

	Fully-justified text		Left-justified text	
	Single column	Double columns	Single column	Double columns
Line-final fixation (ms)	204	208	200	213
	(11)	(11)	(10)	(12)
Accurate line-initial fixation (ms)	234	243	245	241
	(18)	(15)	(18)	(16)
Undersweep-fixation (ms)	192	217	191	216
	(17)	(22)	(15)	(19)

**Table 7:** Fixed effects estimates from LMMs for line-final fixation, accurate line-initial fixations and undersweep-fixations

Dependent measure	*b*	SE	*t*	*P*
Line-final fixation				
Intercept	5.26	0.02	311.72	** < .001**
Text justification	− 0.002	0.009	− 0.28	.778
Column setting	0.05	0.01	4.13	** < .001**
Justification*Column	0.02	0.02	1.28	.202
Accurate line-initial fixation				
Intercept	5.41	0.02	269.32	** < .001**
Text justification	0.01	0.01	0.73	.466
Column setting	0.01	0.01	0.74	.462
Justification*Column	− 0.05	0.02	− 1.93	.054
Undersweep-fixation				
Intercept	5.25	0.02	266.45	** < .001**
Text justification	0.01	0.02	0.63	.527
Column setting	0.13	0.01	9.28	** < .001**
Justification*Column	− 0.01	0.02	− 0.56	.575

Fully-justified condition is the baseline for the analyses of the text justification effect. Single-column condition is the baseline for the analyses of the column setting effect. LMM analyses are based on log-transformed data. Significant terms are marked in bold

For line-final fixation durations, we found a significant main effect of column setting, indicating that line-final fixation durations were significantly longer in the double-column condition than the single-column condition. By contrast, line-final fixation durations were not affected by text justification. The interaction between text justification and column setting was not significant; the influence of column setting on line-final fixation durations was not modulated by text justification. We did not observe significant effects of text justification or column setting for accurate line-initial fixation durations. Similarly, the interaction between text justification and column setting did not reach significance. Together, these results indicate that when return-sweeps were targeted accurately, text format had little influence. Finally, we observed a significant effect of column setting on undersweep-fixation durations such that they were longer in the double-column condition than in the single-column condition. The main effect of text justification and the interaction did not reach significance.

To summarize, compared to the duration of intra-line fixations, the duration of accurate line-initial fixations was significantly longer, while the duration of the line-final and undersweep-fixations was significantly shorter. These findings are consistent with previous studies that have investigated return-sweep fixations in alphabetic languages. Regarding the influence of our manipulations on return-sweep fixation duration, the major effects arose due to column setting with the duration of line-final fixations and undersweep-fixations being significantly longer in the double- than the single-column condition.

## Discussion

In the current experiment, we examined the impact of column setting and text justification when reading multi-line texts among Chinese readers. Our motivation was the need to characterize the general parameters of return-sweeps in Chinese as this language is a non-alphabetic language without inter-word spacing. To date, we have comparatively little knowledge of return-sweep behavior during Chinese reading relative to such behavior in alphabetic languages like English. We also considered that multi-line reading (rather than single-line reading) is very important to investigate since multi-line texts are the types of text that we most often engage with on a daily basis. As such, any differences based on our manipulations may have implications for the way those responsible for the presentation of texts (e.g., publishers) best deliver passages of written information. Finally, this work might also contribute to the development of theoretical models of eye movement control in reading.

The findings from our experiment suggest that differences in column setting and text justification did not negatively influence overall reading in terms of comprehension or reading speed. However, these variables did exert effects in relation to local return-sweep-related behaviors. Specifically, return-sweep and corrective saccades were affected by column setting and text justification, very largely in an additive manner. We replicated previous findings showing that, compared to intra-line fixations, line-final fixations were shorter, accurate line-initial fixations were longer, and undersweep-fixations were shorter. Text justification did not affect return-sweep fixation duration. In contrast, column setting did affect the duration of line-final fixations and undersweep-fixations. The additive and localized nature of these effects alongside minimal disruption to global reading behavior suggests that the influence of column setting and text justification is complex and very probably trade-off against each other to some degree. What is very clear, at least for natural Chinese reading, is that it is not the case that particular combinations of paired rather than single-column settings, or left- compared with fully-justified text formats produce more efficient and effective reading.

### Comprehension accuracy, page reading time, and global measures of eye movements

Recall, in alphabetic languages, although only a very small number of studies have demonstrated format influences on comprehension rates, for those that do report differences, comprehension accuracy was reduced and reading times were longer for fully-justified than left-justified text (e.g., Gregory & Poulton, 1970; Muncer et al., 1986; Sanders & Stern, 1980). It has been argued that the so-called full-justification disadvantage occurred mainly because of the variable and unpredictable word spacing in fully-justified alphabetic texts. Recall, though, that word spacing is absent in written Chinese, and therefore, variability in this regard here was also absent. It is perhaps unsurprising, therefore, that we observed little disadvantage of full justification for comprehension accuracy and the majority of the global measures of eye movements. Thus, it is quite possible that our failure to observe effects occurred because word spacing variability does not arise in fully-justified (as well as left-justified) Chinese texts. Further and similarly, we found comprehension accuracy and global measures of eye movements were also not affected by column setting. That is to say, the act of splitting single-column texts into double columns, thereby reducing line length and increasing line number, did not detrimentally affect overall reading performance. We will return to this issue when we discuss the results of return-sweeps and corrective saccades.

### Return-sweep and corrective saccades

We observed significant effects of text justification for return-sweep and corrective saccades in Chinese multi-line reading. Return-sweep launch position and undersweep-fixation locations were closer to line margins in the reading of left-justified texts than for fully-justified texts. Consistently, more corrective saccades occurred in the left-justified condition than in the fully-justified condition. These findings might suggest that participants experienced greater difficulty processing information close to line margins and relied more on foveal processing when texts were presented with left justification. Such difficulty might arise from the irregularity of line length. Variability in line length in left-justified text means that if readers are to precisely position their eyes at line beginnings, then they must compute return-sweep saccade metrics that are different for each line of text (due to differences in line end position). This is not the case for fully-justified text. A further reason why this effect occurred might be that our Chinese readers were less familiar with left-justified than fully-justified texts as Chinese is ordinarily presented fully-justified with even right margins. A final factor that may also have contributed to return-sweep text justification effects is the extent of visual crowding. When visual crowding is reduced, an increase in the visual span is observed, that is, an increase in the number of characters that can be reliably identified without moving the eyes (Legge et al., 2007; Wang et al., 2014). Although the inter-character spacing adjustments we made to attain text justification in our Chinese stimuli were minimal and minor, it remains the case that in the fully-justified condition the characters were very minimally more horizontally distributed on average than in the left-justified condition. It is possible that increased inter-character spacing might have provided a very small, but arguably sufficient, reduction in visual crowding in the fully-justified condition to have enabled readers to identify characters further to the right of the point of fixation than in the left-justified condition, and thus, reduced the need to foveate further to the line extremes.

More generally, the return-sweep findings are in line with previous studies (e.g., Heller, 1982; Parker & Slattery, 2021; Parker et al., 2019a, 2019b). For example, Heller (1982) found that when text was rated as difficult, more corrective saccades occurred following return-sweeps. Netchine et al. (1983) found that when reading text in a non-native language, both children and adults made more corrective saccades following return-sweeps. Parker et al., (2019a, 2019b) found that, compared to adults, children, who were less skilled readers, tended to launch and land a return-sweep from a position closer to line margins and they produced more corrective saccades. Together, these findings suggest that participants are less able to utilize parafoveal processing to encode information at line margins when they are less skilled or they are reading texts presented in unfamiliar text format, or in a non-native language. Thus, readers in the current study may have utilized parafoveal information to a lesser extent, as evidenced by launch sites closer to the right margin for the less-familiar left-justification condition relative to the fully-justified format. This, in turn, may have led to the need to target the eyes closer to the left margin for new lines of text.

As we predicted, we found that column setting affected return-sweep and corrective saccades. Our finding that return-sweep landing positions were further from the margin and that readers made more corrective saccades in the single-column than the double-column were consistent with existing typical line length effects (e.g., Heller, 1982; Hofmeister et al., 1999; Parker & Slattery, 2021; Parker et al., 2019a, 2019b; Vasilev et al., 2021). As our eyes move through lines of text, saccadic errors frequently occur due to systematic and random errors (McConkie et al., 1988). Targeting a return-sweep to the beginning of a new line from a further distance (i.e., for long relative to short lines) would likely produce increased saccadic error and therefore more corrective saccades would be required to relocate the point of fixation to the intended location. This most likely accounts for why more corrective saccades were needed for single-column presentations relative to multiple-column presentations. Furthermore, we found that return-sweep launch position was further from the right margin of lines in the single-column condition than the double-column condition. This might be because such behavior would reduce the probability of undershoot errors and increase the chance that a reader would make an accurate line-initial fixation after a return-sweep.

Returning to our results pertaining to overall reading performance, to reiterate, this was not affected by column setting. This finding is directly related to our findings that reduced line length contributed to reduced frequency of corrective saccades while the increased number of lines of text naturally required more return-sweeps. It appears, therefore, that there were cost–benefit trade-offs in the effects we obtained. To be clear, when the lines of text were shorter, the benefits of reduced return-sweep undershoot errors on reading appear to have been counteracted by the need to make additional numbers of return-sweeps due to increased line number and reduced availability of parafoveal and peripheral visual information.

### Intra-line fixation durations and return-sweep fixation durations

First, we will consider return-sweep fixations relative to intra-line fixations. Compared to intra-line fixations (223 ms), line-final fixations were shorter (206 ms), and accurate line-initial fixations were longer (235 ms). These results are consistent with those observed in alphabetic languages (e.g., Abrams & Zuber, 1972; Kuperman et al., 2010; Parker & Slattery, 2019; Parker et al., 2017, 2020, 2023). Assuming that these results reflect visual and cognitive processes at mid-line, line-final and line-initial positions and given that the fundamental format characteristics of text across alphabetic and character-based languages are comparable (horizontal text lines read from left to right), the consistency of effects across orthographies is perhaps not surprising. As reviewed in the Introduction, shorter line-final fixations likely arise due to the absence of parafoveal information to process beyond right line margin (thereby making parafoveal processing unnecessary during this fixation). Additionally, to some extent at least, ongoing linguistic processing must be temporarily paused with the primary task during line final fixations being to program a return-sweep to the next line (in order that the next portion of linguistic information might be delivered by the visual system to the language processing system). Of course, it is not clear which of these explanations is more appropriate, or even whether they are mutually exclusive. To us, it seems likely that both these aspects of processing contribute to these effects. Furthermore, we consider that both these accounts might provide rationale for the inflated accurate line-initial fixations. These increased fixation durations are likely to arise jointly due to a temporary interruption to ongoing processing over text lines (Kuperman et al., 2010; Pynte & Kennedy, 2006; Rayner, 1978) and a lack of parafoveal pre-processing for line beginning information (Parker & Slattery, 2019; Parker et al., 2017, 2019a, 2019b; Rayner, 1977).

Undersweep-fixations were inaccurate line-initial fixations that were immediately followed by a corrective saccade. As predicted, undersweep-fixations were shorter than intra-line fixations (199 ms vs. 223 ms). While for some time it was assumed that lexical processing did not occur during undersweep-fixations, recent studies have demonstrated that undersweep-fixations benefit both the processing of the undershot words and the subsequent reading of the words on which mislocated fixations were made (Parker & Slattery, 2019; Parker et al., 2020; Slattery & Parker, 2019). It is the case that fixation durations in Chinese reading are longer than those in alphabetic language reading (see Liversedge et al., 2016, 2024), and in line with this observation, the undersweep-fixations observed in the current study were longer than those reported for alphabetic languages (199 ms vs. 130 ms ~ 170 ms). Given this comparability, it seems plausible that ‘undersweep pre-processing benefit’ effects might exist in Chinese multi-line reading as they do in English reading. Post hoc analyses showed that first fixation durations and gaze durations in the initial interest area following a return-sweep were significantly shorter when there was an undersweep-fixation compared to when there was no undersweep-fixation (first fixation duration: *b* =  − 0.16, SE = 0.008, *t* =  − 21.35, *p* < 0.001; gaze duration: *b* =  − 0.27, SE = 0.009, *t* =  − 28.9, *p* < 0.001). Thus, here we provide evidence for the first time for ‘undersweep pre-processing benefit’ effects in Chinese multi-line reading.

Next, we discuss the influence of our manipulations of text justification and column setting on return-sweep fixations. Line-final fixation durations did not differ between the left-justified condition and the fully-justified condition. By contrast, as discussed earlier, return-sweep launch position (corresponding to the location of a line-final fixation) was closer to the right edge of the line final character (and therefore closer to the initial character on the new line) in the left-justified condition than in the fully-justified condition. These results together suggested that text justification mainly affects decisions about where to position the eyes in reading rather than when the eyes should move in relation to the termination of the line-final fixation immediately preceding a return-sweep. Presumably, processing associated with return-sweep computations is comparable in justified and non-justified text situations.

Given that return-sweep target distance was further in the single-column than the double-column condition, we tentatively predicted that line-final fixations might have a correspondingly increased duration under the assumption that a more distant saccadic targeting computation might be more costly than a less distant saccadic targeting computation. However, contrary to our prediction, line-final fixations were longer in the double-column condition where return-sweep target distance was shorter. We speculate this may be due to differences in strategic saccadic targeting when readers processed texts in single-column and double-column format. In reading single-column texts, readers might adopt a more “risky” return-sweep strategy. Given the increased extent of a return-sweep in single-column text, an undersweep-fixation is quite likely regardless, and therefore rapid approximate targeting might occur. In contrast, in relation to shorter return-sweeps for two-column text presentations, there may be an increased likelihood of accuracy in targeting, and therefore, readers take more time in making this commitment. Of course, this suggestion is speculative and more empirical work is necessary before any firm conclusion can be formed.

While undersweep-fixation durations were not affected by text justification, we did obtain significant effects of column setting such that undersweep-fixations were shorter in the single-column condition than in the double-column condition. The undersweep-fixation location was closer to the line left margin in the double-column condition than in the single-column condition, meaning that smaller undershoot errors occurred in the former. In non-reading tasks, the latency of corrective saccades appears to be determined by undershoot error size (e.g., Becker, 1976) such that for smaller undershoot errors, the latency of corrective saccades is likely to be longer. In line with these results, therefore, it is possible that the smaller undershoot error that occurred in the double-column condition may have reflected the increased time required to a program a corrective saccade. When undershoot error size was smaller, perhaps a more sophisticated computation was required leading to longer undersweep-fixations. Quite why this might be the case, however, is at present unclear.

To summarize, we examined the effect of column setting and text justification on reading processes for multi-line texts in a logographic writing system. Our results demonstrated that the way in which the text was presented did not result in global differences in reading efficiency and effectiveness (e.g., reading speed and comprehension accuracy), and effects were confined to return-sweep eye movement behavior (e.g., return-sweep and corrective saccades). Overall, our results are similar to those reported in a recent study that examined whether typesetting factors across line boundaries influence the reading of multi-line text in English (Parker et al., 2023). In their study, the position of low-frequency words across line boundaries (either at the start or the end of a line) had little impact on global reading performance (i.e., reading time and comprehension) but did have robust effects on local eye movements (e.g., return-sweeps). Our results indicate that there were trade-offs in fine-grained aspects of eye movement control around return-sweeps during multi-line text reading. When return-sweeps were longer under single-column format conditions, line-final and undersweep-fixations were shorter, but more corrective fixations were made. In contrast, when return-sweeps were shorter, under double-column formats, line-final and undersweep-fixations were longer but fewer corrective fixations were made. Further, at a broader level, our study provides evidence that return-sweep processing is similar during the reading of logographic compared with alphabetic scripts, suggesting that return-sweep programming is not script specific. Our findings contribute to existing knowledge of an under-researched aspect of eye movements in reading and might assist researchers in progressing holistic theoretical models of reading.

## Footnotes

1 In a set of additional analyses, we examined whether the effect of text format on return-sweep fixation duration differed from intra-line fixation duration. In the model with an interaction between fixation type and text justification, we observed no significant interaction indicating that the influence of text justification on return-sweeps fixation duration is similar to intra-line fixation duration. In contrast, in the model with an interaction between column setting and fixation type, we observed a significant interaction suggesting that column setting influenced return-sweep fixations more compared to intra-line fixation duration.

## Declarations

### Open practices statement

All analysis scripts, research data and materials are available at https://osf.io/ezafv/?view_only=3ef000bd8ea64f479338beb638902261. This study was not pre-registered.
